# Can’t Sleep

**Published:** 1870-03

**Authors:** 


### CAN'T SLEEP.

WHILE at the hotel of Herr Lang, an estimable gentleman, at Heidelberg, Germany, in 1868, an eminent professional man of New York had taken rooms, the object of his visit being to get some sleep. He called in medical advice shortly after his arrival ; assurance was given him that at least some benefit might be derived from a plan of treatment proposed. The next morning the patient was a new man ; he had had a delightful sleep of one whole night. He was in ecstasies ; and declared with considerable enthusiasm that it was worth a voyage across the Atlantic to have one night's sweet sleep.

Several years ago it was related in these pages that a fine-looking old man, who had retired from business many years before with a large fortune, declared to the writer that he never slept more than two or three hours during the night, and often not that much, and that he offered to give half his fortune, or to begin the world anew, without a dollar, if he could only sleep as well as when he was a young man. Shortly after, he had stolen from him something over six hundred and fifty thousand dollars ; the efforts made to obtain some clew of the robbers, extending over some months, requiring a great deal of personal exertion, mental and physical, so acted upon mind and body, that he began to sleep as well as anybody, especially when he had succeeded in getting back his money, with the exception of the fraction of the hundred thousand.

To-day, a man of wealth and prominence said, on his son being inquired for, a fine-looking young man, of six feet, about twenty-five, free from all the vices of the times, and a creditable member of the church, " He has gone abroad. He couldn't sleep."

What a happy set of fellows our farmers and mechanics ought to be, our founderymen, our masons and carpenters, and hod-carriers! Why, they can sleep twelve hours at a stretch, if daylight did not come, and the need of daily toil did not hurry them from their beds of delicious repose. To make a trip across the Atlantic; to be willing to give hundreds of thousands of dollars to get some sleep, how miserable such must be; and yet the day-laborer envies the rich man his money and his leisure almost as much as the rich man envies their sleep and appetite. If men would only look at the compensations of life; see how evenly they were adjusted, much more evenly than the majority imagine, there would be more thankfulness than there is for what we enjoy, and greater contentment with our lot. The rich can't get to sleep; the poor can't wake up; that is, would oversleep themselves, if their necessities did not wake them.

These things are fully considered in our book on sleep and in previous numbers of this *Journal;* but the reader should carry away with him two practical truths: As a vigorous and healthful appetite and digestion are the inevitable results of labor performed day after day, a labor which secures an encouraging remuneration, so healthful, sweet, delicious sleep can only come as a consequence of compulsory mental or physical effort.

The best position in which to go to sleep is on the right side; the heart being on the left, it has greater freedom of action than when the weight of that part of the body is on it.

Any remaining food in the stomach passes out of it, as the contents of a bottle are passed out of its mouth if turned upside down, as the exit of the stomach is at that part; but if resting on the left side, the food has to be brought up the whole length of the stomach, as water is drawn up from a well, and the effort necessary to this may prevent sleep. Those who take anodynes to promote sleep, instead of procuring it by moderate bodily activities in the open air, make a dangerous experiment.

Sleep is sometimes interfered with by coldness of the extremities in old and young of a feeble circulation; such should wear at night good warm woolen drawers, until, by obtaining more vigorous health, the cause of coldness is removed; and such should not rest satisfied until the drawers can be dispensed with, because the more clothing worn at night, the more will be required in the day-time; the proper and only healthful source of comfortable animal heat is a vigorous digestion.

